# Mediation Analysis Between Brain Age, Disease-Modifying Factors, and Disability and Cognitive Performance in Multiple Sclerosis

**DOI:** 10.1212/WNL.0000000000218161

**Published:** 2026-06-17

**Authors:** Lonneke Bos, Alle Meije Wink, James H. Cole, Giuseppe Pontillo, Bastiaan Moraal, Joep Killestein, Brigit A. de Jong, Bernard M.J. Uitdehaag, Frederik Barkhof, Menno M. Schoonheim, Bas Jasperse, Eva M.M. Strijbis

**Affiliations:** 1 MS Center Amsterdam, Radiology and Nuclear Medicine, Amsterdam Neuroscience, Amsterdam UMC location Vrije Universiteit, the Netherlands;; 2 UCL Hawkes Institute, Department of Computer Science, UCL, London, United Kingdom;; 3 UCL Dementia Research Centre, Queen Square Institute of Neurology, UCL, London, United Kingdom;; 4 UCL Queen Square Institute of Neurology and Hawkes Institute, University College London, United Kingdom;; 5 MS Center Amsterdam, Neurology, Amsterdam Neuroscience, Amsterdam UMC location Vrije Universiteit, the Netherlands; and; 6 MS Center Amsterdam, Anatomy and Neurosciences, Amsterdam Neuroscience, Amsterdam UMC location Vrije Universiteit, the Netherlands.

## Abstract

**Background and Objectives:**

The brain-predicted age difference (brain-PAD) is considered a marker of neurodegeneration in people with multiple sclerosis (pwMS) and is associated with greater disability and cognitive impairment. However, the impact of disease-modifying factors (DMFs) on brain-PAD remains unknown, as does the extent to which their effect on disability and cognition is mediated through brain-PAD. The goal of this study was to investigate this in pwMS using a same-age cohort to eliminate calendar age as a confounding factor.

**Methods:**

Brain age was determined using brainageR from 3-dimensional T1-weighted MRI scans, and brain-PAD was calculated as the difference between predicted and chronological age. Disability was evaluated with the Expanded Disability Status Scale (EDSS), the 9-hole peg test (9HPT), and timed 25-foot walk test (T25FWT). Cognitive function was assessed using the Minimal Assessment of Cognitive Function in MS battery and converted to *Z*-scores. DMFs included lifetime smoking, alcohol consumption, physical activity, diet, leisure, and educational level, as well as body mass index (BMI) at 18 years. The effect of DMFs on brain-PAD was examined using linear regression, adjusting for sex. Mediation analyses were performed to investigate to what extent brain-PAD is a mediator of DMF effects on disability and cognition.

**Results:**

The study included 117 healthy controls (mean age 52.8 ± 1.1 years, 74.3% female) and 242 pwMS (mean age 52.8 ± 0.9 years, 70.6% female), with a median disease duration of 15.2 years (interquartile range [IQR] 8.2–24.5) and a median EDSS score of 3.5 (IQR 2.5–4.0). Smoking (β = 0.20, *p* = 0.002), alcohol consumption (β = 0.18, *p* = 0.007), and BMI at 18 years (β = 0.15, *p* = 0.021) were associated with a higher brain-PAD, whereas higher physical activity (β = −0.13, *p* = 0.041) was linked to a lower brain-PAD. Mediation analysis demonstrated an indirect effect of smoking and alcohol on EDSS score (β = 0.039, *p* = 0.003; β = 0.039, *p* = 0.009), 9HPT (β = 0.057, *p* < 0.0001; β = 0.055, *p* = 0.004), T25FWT score (β = 0.045, *p* = 0.009; β = 0.044, *p* = 0.013), and cognitive performance (β = −0.066, *p* = 0.0004; β = −0.060, *p* = 0.016). Physical activity demonstrated an indirect effect of better performance on EDSS score (β = −0.027, *p* = 0.032) and cognitive performance (β = 0.042, *p* = 0.034).

**Discussion:**

DMFs unrelated to MS, particularly smoking, alcohol use, and BMI at 18 years, accelerate brain aging. Brain-PAD mediates these effects and contributes to worsening disability and cognition, underscoring the potential of lifestyle interventions to mitigate neurodegeneration and preserve function in pwMS.

## Introduction

Age and neurodegeneration are associated with disease progression in multiple sclerosis (MS), a chronic inflammatory demyelinating disease of the CNS,^1^ influencing both disability and cognitive decline.^2^ Atrophy measures, particularly brain volume loss, have proven to be valuable biomarkers for disease progression.^3,4^ However, quantifying atrophy in individual patients remains challenging because of variability in normal aging and technical factors.^5,6^

In recent years, brain age estimation has emerged as a neuroimaging biomarker of neurodegeneration.^7-9^ Machine learning models trained on healthy individuals can estimate a person's age from conventional brain MRI scans.^10^ Deviation from the healthy norm is quantified using the brain-predicted age difference (brain-PAD), obtained by subtracting the chronological age from the brain-predicted age. In people with MS (pwMS), brain-PAD was found to be higher than in healthy controls (HCs). Longer disease duration is related to a higher brain-PAD, and a higher brain-PAD has been associated with greater disability and cognitive impairment, supporting its relevance as a marker for neurodegeneration and disease severity in MS.^11-15^

Brain aging in MS shows substantial interindividual variability. Some individuals show signs of accelerated brain aging, suggesting neurodegeneration earlier or more severely than expected, while others of similar chronological age show relatively preserved brain structure, suggesting decelerated brain aging.^13^ This variability cannot be fully explained by age alone, drawing attention to other potential explanatory factors.^16^ Among these, disease-modifying factors (DMFs), particularly lifestyle-related behaviors, are of growing interest. A UK Biobank study showed that brain aging is associated with smoking and alcohol use^17^ in healthy people. In MS, behaviors such as smoking, physical inactivity, and poor dietary intake have been linked to worse disability progression.^18-20^ Nevertheless, the role of DMFs in shaping brain aging trajectories in MS, as reflected in MRI-based measures such as brain-PAD, remains unclear and is unexplored in existing literature.

To disentangle the effect of DMFs from age-related changes, we used a cohort of people with MS, all born in 1966, to investigate the effect of DMFs on brain-PAD. Subsequently, we aimed to assess the extent to which these DMF-related changes in brain-PAD are associated with disability and cognitive function.

## Methods

### Study Population

This study included HCs and patients of the Project Y cohort,^21^ which is a population-based cross-sectional birth-year cohort of pwMS, aiming to identify factors explaining phenotype variability in MS. To be considered eligible for participation in Project Y, patients were required to meet all of the following criteria: (1) born in the Netherlands in 1966; (2) currently living in the Netherlands; (3) diagnosis of MS according to the 2010^22^ or 2017^23^ McDonald criteria. HCs had to meet the following criteria: (1) born in the Netherlands between 1965 and 1967; (2) currently living in the Netherlands; (3) no history of MS. After screening, a total of 367 pwMS were included. Of these, 271 pwMS and 125 HCs visited our center for a full day of testing, whereas the remaining participants received a home visit. Inclusion criteria for this particular research question were a diagnosis of clinically definite MS, availability of high-quality MRI scans, disability evaluations, cognitive tests, and completion of a set of in-house developed questionnaires. HCs who had undergone MRI and cognitive assessments were also included. Exclusion criteria were missing data on the clinical measures or DMFs, or no availability of MRI scans of sufficient quality. All examinations were performed during a 1-day study visit, between December 2017 and January 2021.

### Standard Protocol Approvals, Registrations, and Patient Consents

The Medical Ethical Committee of the Amsterdam UMC, location VUmc, approved the Project Y protocol. Written informed consent was obtained from all participants at inclusion, according to the Declaration of Helsinki. The study is registered at the Netherlands Trial Register (NL6362).

### Disease-Modifying Factors

DMFs were defined as lifestyle factors and sociodemographic characteristics that have the potential to influence disease progression or brain structure in pwMS. Participants were asked to complete a set of in-house developed questionnaires, after the 1-day study visit. The questionnaire contained questions on lifestyle factors, such as diet, physical activity, smoking, alcohol, and leisure, early and later in life. Sociodemographic factors such as level of education, current body mass index (BMI), and BMI at 18 years were also assessed. The lifestyle factors were assessed following the same approach as previously described.^24^

Dietary intake was assessed by asking participants “how many times a week did you eat the following foods when you were around 10, 30, and 50 years old” (“vegetables,” “fruit,” “red meat,” “oily fish,” “whole grain bread,” and “fast food/snacks/candy”). For each of the dietary components, participants were categorized into tertiles based on their reported intake. The diet quality score for each age was determined by rewarding points for a high intake of fruit and vegetables, oily fish, and whole-grain bread (e.g., participants in the lowest tertile received a score of 1 and participants in the highest tertile received a score of 3). For high intake of red meat and fast food/snacks/candy, scores were reversed and participants in the lowest tertile received a score of 3 and those in the highest tertile a score of 1. The total diet quality score at each age was calculated by adding all component scores and ranged from 6 to 18. The overall diet quality score was computed by summing the 3 diet quality scores.

Physical activity was assessed by asking the following questions: “how often, in a normal week, did you participate in intensive activities (e.g., running, soccer, hockey) at the age of 10, 30 or 50 years for at least 10 minutes?” and “at the age of 10, 30 of 50 years, how often, in a normal week, did you participate in moderately intensive activities (e.g., badminton, tennis, brisk walking) for at least 10 minutes?” The cumulative physical activity at each age was calculated by summing the number of intensive activities and moderately intense activities. The overall physical activity score was then computed by summing the 3 physical activity scores.

The amount of lifetime smoking was quantified using “packyears,” that is, years smoked times the amount of packs per day.

Alcohol intake was assessed at approximately 20 years, 40 years, and the current age. Participants reported the frequency of alcohol consumptions per week and the average number of alcoholic drinks consumed per day at each age. Weekly alcohol consumption was estimated by multiplying frequency and quantity at each age. The total alcohol degree was then computed as the sum of these 3 values. If participants indicated lifetime abstinence from alcohol, the alcohol degree was set to 0.

Leisure was assessed based on participants' reported frequency of engaging in activities such as “reading books,” “reading magazines,” “creating or appreciating art,” “writing,” “making music,” or “engaging in hobbies,” in their early 20s. Each activity was rated on a 6-point scale, where higher scores indicated more frequent participation. The leisure score was calculated by summing the values of the activities.

Other DMFs included BMI at 18 years, which was calculated by dividing weight at the age of 18 by the square of the height. The level of education was scaled between 1 and 7, where 1 = no education; 2 = primary education; 3 = primary education completed and further education <2 years; 4 = prevocational secondary education; 5 = vocational secondary education; 6 = secondary education (higher general secondary education and preuniversity education); and 7 = university/postdoctoral education.

### Disability and Cognitive Measures

The Expanded Disability Status Scale (EDSS),^25^ the 9-hole peg test (9HPT),^26^ and the timed 25-foot walking test (T25FWT)^27^ were used to assess disability. For the 9HPT, the average of the 2 dominant hand trials and 2 nondominant hand trials were used. The T25FWT score was calculated by averaging the 2 performed trials. Cognitive functioning was assessed using a test battery based on the Minimal Assessment of Cognitive Function in MS (MACFIMS). The battery consists of multiple tests assessing verbal fluency (Controlled Oral Word Association Test), visuospatial perception (Benton Judgment of Line Orientation Test), visuospatial memory (Brief Visuospatial Memory Test-Revised), verbal memory (Verbale Leer- en Geheugentaak, the Dutch version of the California verbal learning test), information processing speed (Symbol Digit Modalities Test), and executive functioning (Delis-Kaplan Executive Function System sorting test). All scores were corrected for effects of age, sex, and years of education in the HC group, using linear regression models, to standardize the neuropsychological test scores. Corrected scores were converted to *Z*-scores for all participants based on the means and standard deviations of the HC group described in the study by Loonstra et al.^21^

### MRI

All participants underwent 3T MRI of the brain, using the same scanner (3T, Discovery MR750; GE, Milwaukee, WI). Brain age was determined using a 3-dimensional (3D) T1-weighted (T1w) fast spoiled gradient-echo sequence (repetition time 8.2 milliseconds, echo time 3.2 milliseconds, flip angle 12°, 1 × 1 × 1-mm voxel size, acquisition direction: sagittal). For T2 lesion quantification, a 3D fluid-attenuated inversion recovery (FLAIR) image (repetition time 8,000 milliseconds, echo time 125 milliseconds, inversion time 2,350 milliseconds, 1.2 × 1 × 1-mm voxel size, acquisition direction: sagittal) was used.

### Brain Age Prediction

Brain age was predicted using publicly available software (brainageR), which uses raw 3D T1w-MRI scans to predict brain age using Gaussian process regression (GPR).^28^ brainageR was trained on data from 3,377 healthy individuals (mean age ± SD 40.6 ± 21.4 years; range 18–92 years) across 7 publicly available data sets and tested on an independent cohort of 857 healthy individuals (mean age ± SD 40.1 ± 21.8 years; range 18–90 years).^7,10,29^ All participants were confirmed healthy, based on local study data.

Our input T1w scans were preprocessed with SPM12^30^ for segmentation and normalization to a template; then converted into feature vectors representing gray matter, white matter, and CSF, which were masked using a brainageR-specific template; and reduced by principal component analysis. The resulting principal components were used as input to the GPR model to predict brain age. Brain-PAD was determined by subtracting brain-predicted age from chronological age.

### MRI Processing

Raw 3D T1w-MRI scans were bias field–corrected using Advanced Normalization Tools,^31^ followed by skull stripping for T1w and 3D-FLAIR images using HD-BET.^32^ After linearly registering FLAIR to T1w, lesion segmentation was performed using nicMSlesions.^33^ To avoid potential variation in tissue segmentation due to MS lesions,^34^ the resulting lesion masks were manually corrected and used for T1 lesion filling using the Lesion Segmentation Tool.^35^ The manually corrected masks were used to determine T2 lesion volume (T2LV) with fslstats, from the FMRIB Software Library.^36^ The recon-all pipeline of FreeSurfer 7.1.1^37^ was used to automatically perform whole-brain tissue-type segmentation on the lesion-filled 3D T1w images. Subsequently, the brain parenchymal fraction (BPF) was obtained by dividing the total brain volume excluding ventricles (BrainSegVolNotVent) by the estimated total intracranial volume.

### Statistical Analysis

Statistical analyses were performed using R Statistical Software (version 4.3.2; R Foundation for Statistical Computing, Vienna, Austria). Variables were tested for normality by histogram and QQ-plot inspection. The 9HPT and T25FWT test scores were log-transformed. A 2-sided *p* value of <0.05 was considered significant. We did not perform multiple comparison corrections because of the exploratory analysis of the study. To investigate whether there is a potential bias toward individuals with a milder disease course, the differences in clinical measures and DMFs between pwMS with a home visit and those with a whole-day hospital visit were analyzed. To guide covariate selection, a directed acyclic graph (DAG) was constructed for each association of interest, representing the assumed individual causal relationships with sex as a confounder.

#### Regression Analysis of DMFs

To investigate interaction terms between DMFs for pwMS, we conducted a Pearson correlation matrix using standardized values of “alcohol,” “BMI at 18 years,” “diet,” “disease duration,” “educational level,” “leisure,” “physical activity,” “sex,” and “smoking.” A heatmap was generated to visualize the correlation.

To assess the relation between each different DMF and brain-PAD, we conducted a univariate linear regression, adjusted for sex, both for controls and for pwMS. The relation between the current use of disease-modifying treatment (DMT), the current DMT efficacy, lifetime highest efficacy DMT, and duration of treatment weighted to efficacy classes, and brain-PAD, adjusted for sex, was also investigated. As additional analysis, we repeated these models using BPF and T2LV as the outcome measure, to compare the DMF effects on conventional volumetric measures, for pwMS.

#### Mediation Analysis

For the DMFs that showed a significant association with brain-PAD in the univariate analysis and the clinical measures that were significantly associated with brain-PAD (eFigure 1),^15^ we conducted a mediation analysis to examine whether brain-PAD mediated the relationship between individual DMFs and the clinical outcome measures. The clinical outcome measures were EDSS, 9HPT, T25FWT, and cognitive performance, which were assessed by averaging the *Z*-scores of the tests from the MACFIMS battery. For each DMF, 2 linear models were specified:

Brain‐PAD ∼ DMF+sex

Clinical outcome ∼ Brain‐PAD+DMF+sex

The DMF was specified as the independent variable and brain-PAD as the mediator. Mediation analysis were conducted using the *mediation* package in R,^38^ where a regression-based approach was applied following the counterfactual framework, which was tested using nonparametric bootstrapping with 5,000 simulations to estimate the indirect (mediated), direct, and total effects and their corresponding CIs. The analysis indicates the *indirect effect* of the DMF through brain age on the clinical outcome measure (average causal mediation effect) and a *direct effect* independent of brain age (average direct effect). The *total effect* represents both the direct and indirect effect together.

### Data Availability

Data may be shared (pseudonymized) at the request of any qualified investigator for purposes of replicating procedures and results.

## Results

### Demographics

Characteristics for the HC and different MS phenotypes are summarized in Table 1. From the original Project Y cohort, we excluded 125 of 367 pwMS and 8 of 125 HCs because of missing or insufficient quality data, or because data were unavailable for participants who underwent a home visit. The final cohort in this study included 117 HCs (75% female) and 242 pwMS (71% female), comprising 153 with relapsing-remitting MS, 55 with secondary progressive MS, and 34 with primary progressive MS. For HCs, the mean age was 52.8 (±1.2) years, the mean brain age was 47.2 years (±6.7), and the mean brain-PAD was −5.6 years (±5.7). For pwMS, the mean age was 52.8 years (±0.9), the mean brain age was 56.6 years (±8.7), the mean brain-PAD was +4.0 years (±8.7), and the median disease duration was 15.2 years (interquartile range [IQR] 8.2–24.5). The median EDSS score was 3.5 (IQR 2.5–4.0).

**Table 1:** Demographic, Clinical, and MRI Characteristics in Patients With MS and HCs

	HC (n = 117)	All (n = 242)	RRMS (n = 153)	SPMS (n = 55)	PPMS (n = 34)
Demographical features					
Age, y, mean (SD)	52.8 (1.2)	52.8 (0.9)	52.8 (0.9)	52.7 (0.8)	53.1 (0.9)
Sex (female), n (%)	87 (74.3)	172 (70.6)	124 (81.0)	32 (58.2)	16 (47.1)
Level of education, median (IQR)^a^	6 (5–6)	6 (5–6)	6 (5–6)	5 (5–6)	6 (5–6)
Clinical features					
Disease duration since onset, y, median (IQR)		15.2 (8.2–24.5)	14.6 (8.0–24.3)	21.7 (15.9–28.4)	8.5 (5.0–14.9)
DMT ever (yes), n (%)		166 (68.6)	110 (71.8)	45 (81.2)	11 (32.4)
Current DMT efficacy, n (low/middle/high/none)		49/8/8/177	40/5/5/103	8/3/2/42	1/0/1/32
Brain age, y, mean (SD)	47.2 (6.7)	56.6 (8.7)^b^	55.8 (9.1)	60.0 (8.0)	56.7 (6.8)
Brain-PAD, y, mean (SD)	−5.6 (5.7)	4.0 (8.7)^b^	2.9 (9.1)	7.2 (7.8)	3.6 (6.6)
Brain parenchymal fraction, mean (SD)	0.737 (0.03)	0.712 (0.05)^b^	0.713 (0.03)	0.710 (0.04)	0.712 (0.05)
Lesion volume, mL, median (IQR)	0.04 (0.0002–0.060)	6.4 (2.9–13.2)^b^	5.7 (2.8–11.6)	9.9 (3.9–19.8)	6.8 (2.4–12.9)
Clinical scores					
EDSS, median (IQR)		3.5 (2.5–4.0)	3.0 (2.0–4.0)	5.5 (3.75–6.25)	4.0 (3.5–6.0)
9HPT, s (left and right), median (IQR)^c^		21.6 (19.4–24.9)	20.5 (18.9–23.2)	24.2 (21.5–35.7)	22.9 (21.7–26.0)
T25FW, s, median (IQR)^c^		4.8 (4.2–6.0)	4.5 (4.0–5.3)	6.0 (5.0–9.0)	5.5 (4.8–7.5)
Cognitive scores					
SDMT, mean (SD)	58.6 (8.5)	50.6 (10.8)^b^	52.8 (10.1)	45.8 (9.6)	48.4 (12.9)
COWAT, mean (SD)	13.8 (3.7)	12.0 (4.0)^b^	12.7 (3.8)	10.7 (3.6)	11.3 (4.7)
JLO, mean (SD)	26.5 (3.2)	25.5 (4.1)^b^	25.5 (3.9)	25.5 (5.1)	25.4 (3.1)
D-KEFS, mean (SD)	10.9 (1.9)	9.9 (2.3)^b^	10.1 (2.2)	9.7 (2.4)	9.3 (2.6)
VLGT-r, mean (SD)	10.9 (2.1)	9.7 (2.4)^b^	10.1 (2.1)	9.1 (2.3)	9.3 (2.4)
BVMT-r, mean (SD)	8.8 (1.6)	7.8 (2.1)^b^	8.0 (2.0)	7.4 (2.2)	7.7 (2.7)
Disease-modifying factors					
Packyears, median (IQR)	0 (0–6)	4.3 (0–15.6)^b^	2.4 (0–14.3)	8.5 (0–16.8)	8.9 (0.4–23.1)
Alcohol degree, median (IQR)	8 (5–11)	5 (3–13)	4.5 (1.5–12.5)	5 (3.0–10.5)	8 (3.4–16.6)
Diet, median (IQR)	33 (29–36)	32 (29–35)	32 (29–35)	32 (29–35)	32 (29–36)
Physical activity, median (IQR)	15 (9–20)	14 (9–20)	14 (9–21)	13 (7–19)	14 (12–19)
Leisure, median (IQR)	16 (13–18)	24 (22–27)^b^	24 (22–27)	26 (22–28)	25 (22–27)
BMI at 18 y, mean (SD)	21.2 (2.5)	22.1 (3.8)	21.9 (4.1)	22.6 (2.9)	22.3 (3.2)
BMI current, mean (SD)	25.6 (3.7)^d^	25.6 (4.2)^d^	25.7 (4.2)	25.6 (4.5)	25.2 (3.8)

Abbreviations: 9HPT = 9-hole peg test; BMI = body mass index; BVMT = Brief Visuospatial Memory Test-Revised; COWAT = Controlled Oral Word Association Test; D-KEFS = Delis-Kaplan Executive Function System sorting test; DMT = disease-modifying therapy; HC = healthy control; IQR = interquartile range; JLO = Benton Judgment of Line Orientation Test; MS = multiple sclerosis; PPMS = primary progressive MS; RRMS = relapsing-remitting MS; SDMT = symbol digit modalities test; SPMS = secondary progressive MS; T25FWT = timed 25-foot walk test; VLGT = Verbale Leer- en Geheugentaak.

Table 1 shows the demographic, clinical and MRI characteristics of people with MS.

a The scale used to assess education ranged from 1 (did not finish primary school) to 7 (university or higher) (5 = secondary vocational education (MBO); 6 = higher professional education (HBO)).

b Indicates a statistical difference between the patient group and controls.

c The average of the measurements is shown. Low-efficacy therapy consists of dimethyl fumarate, glatiramer acetate, interferon, and teriflunomide; moderate-efficacy therapy consists of cladribine, fingolimod, methotrexate, and mitoxantrone; high-efficacy therapy consists of alemtuzumab, daclizumab, natalizumab, and ocrelizumab.

d Indicates a statistical difference between BMI at 18 years and current BMI.

The median number of packyears was 4.3 years (IQR 0–15.6), the median alcohol degree was 5 (IQR 3–13), the median score for diet was 32 (IQR 29–35), the median score for physical activity was 14 (IQR 9–20), and the median score for leisure was 24 (22–27). The mean BMI at 18 years was 22.1 (±3.8) and lower than current BMI, which was 25.6 (±4.2). The median level of education was 6 (IQR 5–6).

EDSS scores were higher for pwMS who underwent a home visit; the other features for which we observed no differences between home visit and hospital visit are listed in eTable 1. A DAG illustrating the assumed relationships is shown in eFigure 2.

### Regression Analysis of DMFs

**Figure 1:**
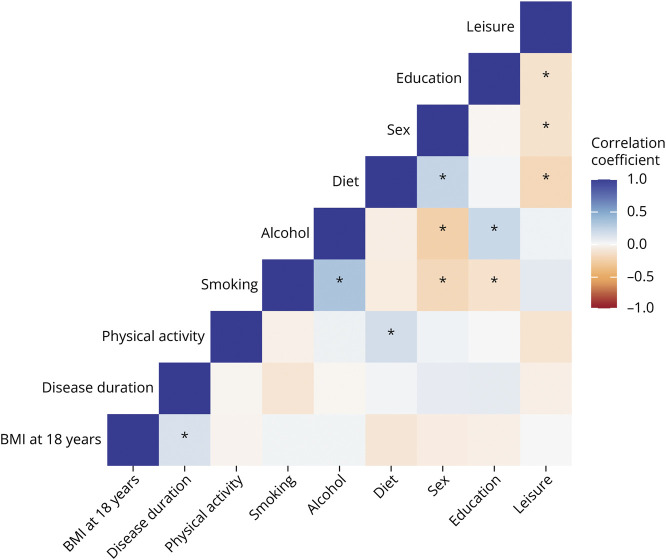
Correlation Matrix of Disease-Modifying Factors Matrix of the Pearson correlation between the disease-modifying factors and demographic factors for only pwMS. Stronger positive correlations are shown in darker blue and stronger negative correlations in darker red. Boxes with a * indicate a significant correlation, with *p* < 0.05. In “sex”: male = 1; female = 2. BMI = body mass index; pwMS = people with multiple sclerosis.

The Pearsons correlation matrix with a heatmap of the different DMFs for pwMS is shown in Figure 1. We found that men reported higher alcohol consumption (β = −0.26, 95% CI −0.32 to −0.19, *p* < 0.0001), more smoking (β = −0.20, 95% CI −0.32 to −0.08, *p* = 0.0016), and worse diet quality (β = 0.22, 95% CI 0.10–0.34, *p* = 0.0004) compared with women. A better diet quality correlated to higher physical activity (β = 0.16, 95% CI 0.10–0.24, *p* = 0.011). A higher BMI at 18 years was associated with a longer disease duration (β = 0.14, 95% CI 0.01–0.26, *p* = 0.034). A higher level of education was associated with more alcohol intake (β = 0.20, 95% CI 0.04–0.29, *p* = 0.0016) and less smoking, as quantified by packyears (β = −0.14, 95% CI −0.26 to −0.02, *p* = 0.028).

**Figure 2:**
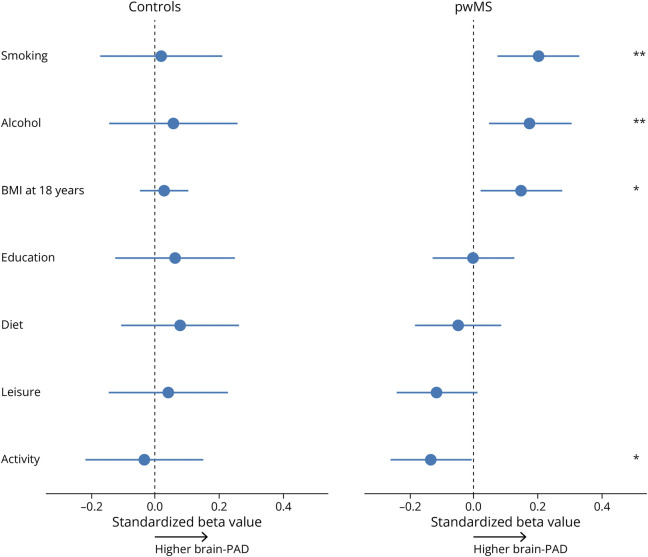
Forest Plot of Linear Regression Between DMFs and Brain-PAD Forest plot showing the standardized β coefficients for the association between DMFs and brain-PAD, adjusted for sex, for controls on the left and pwMS on the right. Positive values indicate a higher brain-PAD (older-appearing brain) while negative values indicate a lower brain-PAD (younger-appearing brain). Error bars represent 95% CIs. *p < 0.05, **p < 0.01. BMI = body mass index; brain-PAD = brain-predicted age difference; DMF = disease-modifying factor; pwMS = people with multiple sclerosis.

Figure 2 shows a forest plot of the standardized β coefficients for the association between the DMFs and brain-PAD, adjusted for sex, for both HCs and pwMS (details shown in eTable 2). We did not find any relation between the DMFs and brain-PAD for HCs. For pwMS, more smoking (β = 0.202, 95% CI 0.08–0.33, *p* = 0.002), alcohol use (β = 0.176, 95% CI 0.05–0.30, *p* = 0.007), and BMI at 18 years (β = 0.149, 95% CI 0.02–0.28, *p* = 0.021) indicated a higher brain-PAD, whereas more physical activity (β = −0.132, 95% CI −0.26 to −0.01, *p* = 0.041) indicated a lower brain-PAD. Analysis on the effect of current DMT efficacy or current DMT use showed no effect on brain-PAD (β = 0.029, 95% CI −0.15 to 0.21, *p* = 0.75; β = 0.045, 95% CI −0.24 to 0.33, *p* = 0.76, respectively). Lifelong highest DMT showed a relation where high-efficacy therapy is associated with a higher brain-PAD (β = 0.144, 95% CI 0.01–0.28, *p* = 0.035). In addition, we found an association of duration of DMT according to efficacy classes with brain-PAD (β = 0.144, 95% CI 0.02–0.27, *p* = 0.025). Univariate linear regression results for the DMFs and BPF only showed a significant relation between BPF and physical activity (β = 0.0056, 95% CI 0.001–0.010, *p* = 0.013). We did not find an effect of the DMFs on T2LV (Table 2).

**Table 2:** Associations of DMFs With BPF and T2LV

DMF	BPF estimate (95% CI)	*p* Value	T2LV estimate (95% CI)	*p* Value
Activity	0.0056 (0.0012 to 0.01)	0.013	−0.017 (−0.039 to 0.005)	0.129
Smoking	−0.0038 (−0.0082 to 0.0007)	0.097	0.012 (−0.0098 to 0.034)	0.274
Alcohol	−0.0035 (−0.008 to 0.001)	0.128	0.0023 (−0.020 to 0.025)	0.837
BMI 18 y	−0.0026 (−0.007 to 0.0018)	0.248	0.018 (−0.0043 to 0.039)	0.115
Diet	0.0026 (−0.0021 to 0.0073)	0.275	−0.011 (−0.034 to 0.012)	0.358
Education	−0.0023 (−0.0067 to 0.0021)	0.302	−0.0004 (−0.022 to 0.021)	0.968
Leisure	0.0014 (−0.003 to 0.0059)	0.523	−0.0084 (−0.030 to 0.013)	0.450

Abbreviations: BMI = body mass index; BPF = brain parenchymal fraction; DMF = disease-modifying factor; T2LV = T2 lesion volume.

Table 2 shows the standardized β coefficients and their corresponding CIs for the association between DMFs and BPF and T2LV, adjusted for sex.

### Mediation Analysis

**Figure 3:**
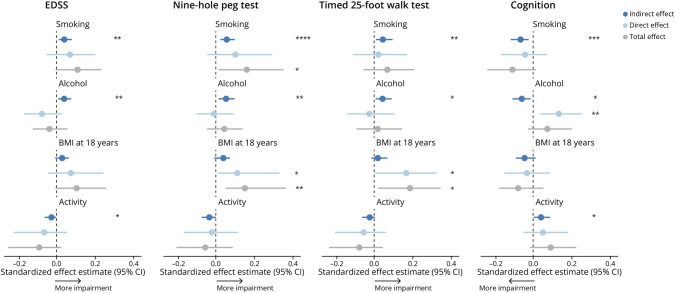
Forest Plots of Mediation Analysis of DMF on Clinical Measures Forest plots showing the relationship between DMFs and clinical outcomes, EDSS, 9HPT, T25FWT, and cognitive performance, using brain-PAD as a mediator. Higher values for EDSS, 9HPT, and T25FWT mean more disability, whereas lower values for cognition mean more cognitive impairment. Standardized effect estimates are reported for the average causal effect (indirect effect), average direct effect (direct effect), and total effect. The model builds on the univariate associations by examining whether the effect of DMFs on clinical outcomes is mediated through accelerated brain aging. Error bars represent 95% CIs. *p < 0.05, **p < 0.01, ***p < 0.001, ****p < 0.0001. 9HPT = 9-hole peg test; BMI = body mass index; brain-PAD = brain-predicted age difference; DMF = disease-modifying factor; EDSS = Expanded Disability Status Scale; pwMS = people with multiple sclerosis; T25FWT = timed 25-foot walk test.

We performed mediation analysis to investigate the indirect, direct, and total effects of the DMFs mediated through brain-PAD on the clinical outcome measures (Figure 3). Our findings report that smoking and alcohol use demonstrated an indirect effect on EDSS (β = 0.039, 95% CI 0.011–0.079, *p* = 0.003; β = 0.039 95% CI 0.008–0.076, *p* = 0.009), 9HPT (β = 0.057, 95% CI 0.024–0.099, *p* < 0.0001; β = 0.055, 95% CI 0.015–0.099, *p* = 0.004), and T25FWT (β = 0.045, 95% CI 0.009–0.096, *p* = 0.009; β = 0.044, 95% CI 0.007–0.093, *p* = 0.013) scores and cognitive performance (β = −0.066, 95% CI −0.117 to −0.025, *p* = 0.0004; β = −0.060, 95% CI −0.108 to −0.013, *p* = 0.016). A total effect of smoking on 9HPT (β = 0.16, 95% CI −0.044 to 0.294, *p* = 0.029) performance was also observed. In addition, alcohol use was associated with better performance of cognitive testing with a direct effect (β = 0.13, 95% CI 0.033–0.254, *p* = 0.009). We found that physical activity demonstrated an indirect effect on EDSS scores (β = −0.027, 95% CI −0.063 to −0.002, *p* = 0.032) and cognitive performance (β = 0.042, 95% CI 0.002–0.088, *p* = 0.034). BMI at 18 years showed no indirect effects, but did show a direct and total effect on 9HPT (β = 0.040, 95% CI 0.011–0.332, *p* = 0.022; β = 0.15, 95% CI 0.052–0.365, *p* = 0.001) and T25FWT (β = 0.17, 95% CI 0.003–0.327, *p* = 0.048; β = 0.19, 95% CI 0.020–0.347, *p* = 0.028). In addition, a visual representation of these mediation analyses is shown in eFigure 3.

## Discussion

In this study, we assessed the effect of DMFs on brain age, aiming to understand the interindividual variability of brain aging in a large cohort of pwMS of the same age (52.8 years). We found that smoking, alcohol consumption, and BMI at 18 years were associated with a higher brain age, and that physical activity was associated with a lower brain age. By contrast, analyses using BPF instead of brain age only showed a positive effect of physical activity. Mediation analysis showed that the effects of smoking, alcohol, and physical activity on clinical outcome measures for disability and cognition were mediated through brain age.

To explore the underlying relationships between the DMFs and sociodemographic factors for pwMS, we conducted a Pearson correlation analysis, visualized using a heatmap. This approach allowed us to examine the multicollinearity that could inform interpretation of the main findings. We found that men reported higher levels of lifetime smoking and alcohol consumption and had worse diet quality, compared with women. Individuals with a better diet quality showed higher values for physical activity. Individuals with a higher level of education had a higher alcohol consumption, but lower values for lifetime smoking. Next, we found that a higher BMI at 18 years was associated with longer disease duration. This is in line with previous research, which has shown that a high BMI during childhood and adolescence increases the risk of developing MS^39,40^ and is associated with higher disability scores and faster disability accumulation,^41^ underscoring the importance of early-life weight management to improve clinical outcomes of MS.

We found that these factors also have a negative effect on brain age for pwMS. Brain age was higher in individuals with more packyears and alcohol consumption and a higher BMI at 18 years. Although a lower brain age was found in people with higher scores for physical activity. We did not find any association between the DMFs and brain age for HCs, indicating that these factors negatively affect the brain and cause more structural damage and advanced brain aging in MS, and not as profound in HCs. This may reflect limited variability in our sample or insufficient power to detect subtle effects, as reported in other studies.^42^ An observed difference between groups was higher packyears in pwMS, which may contribute to the stronger associations with brain age in pwMS than in HCs. To compare our results with a conventional brain volume measure, we repeated the analysis using BPF as the outcome measure. In this study, we did not observe any effects of the DMFs on BPF, except for a protective effect of physical activity. In addition, no effects of the DMFs on T2LV were found. This may reflect the distinct sensitivity of brain age to age-related changes beyond global atrophy. Our findings are in line with the UK Biobank study, suggesting that smoking and alcohol consumption were associated with higher brain age in healthy people.^17^ Adding to this, we showed that a higher BMI during adolescence showed a negative influence because it accelerates brain aging in people with MS. By contrast, more physical activity throughout life was associated with a lower brain-PAD, indicating a protective effect of physical activity on brain aging and structural damage. We did not observe an effect of the current use of DMT or current DMT efficacy on brain aging, when investigating the effect of current DMT efficacy on brain age. We did find a positive relation when investigating lifetime highest efficacy type and treatment duration according to efficacy classes. This may be explained by the fact that high-efficacy DMTs are typically prescribed to individuals with a more aggressive disease course, meaning that brain aging may already be advanced before treatment initiation, leading to a complex relationship between DMT and brain age. In addition, the heterogeneity of treatment histories, limited sample sizes, and the retrospective observational design of the study, rather than a randomized controlled trial, limits the ability to draw conclusions about the effects of DMTs on brain aging. Future studies using longitudinal data could better disentangle the relationship between the use of DMT and brain aging, for example, by examining whether brain age trajectories change after the initiation of treatment.

While understanding the impact of the DMFs on the brain, it is equally important to understand how these effects translate to clinical outcomes. To better contextualize the significance of certain lifestyle choices, we performed mediation analyses to examine the extent to which the influence of DMFs on clinical measures, such as EDSS, 9HPT, T25FWT, and cognition, is mediated through brain age. Our findings show that the effects of lifetime smoking and alcohol consumption are significantly mediated through brain age for all clinical outcome measures, meaning that it does not only affect the brain but also negatively affect the disease course. This aligns with previous studies on lifestyle and neurodegeneration in MS, where smoking and alcohol are associated with more neurodegeneration as indicated by thinning of the macular ganglion cell and inner plexiform layer.^43^ An unexpected finding was that higher alcohol consumption shows a significant positive direct effect on cognition. This could be explained by confounding factors such as social engagement or socioeconomic status, which are often associated with higher alcohol consumption and may independently support cognitive functioning.^44^ We found that brain-PAD mediates the relationship between physical activity and both EDSS score and cognition, showing that physical activity not only is protective for brain damage but also contributes to better clinical outcomes in MS. A higher BMI at 18 years was associated with a direct and total effect on the 9HPT and T25FWT, but not EDSS. This suggests that a high BMI at adolescence has a long-term impact on upper and lower limb motor performance, which may not be fully captured with a more global measure, such as EDSS. This is also shown in a study investigating the effect of BMI across childhood and early adolescence on motor coordination,^45^ which could implicate this as a general developmental effect, which is not specific for MS.

The strength of this study is the unique cohort of pwMS of the same age, which minimizes age-related variability. This comprehensive cohort has extensive data on DMFs throughout life and has extensive measures of disability and cognitive functioning. In addition, no scanner differences are introduced, which eliminates the need to correct for different scanners or software differences. However, our study also has several limitations. First, using a retrospective design introduced the potential for inaccurate recall of the DMFs in early life. Because dietary intake and physical activity were assessed at the ages of 10, 30, and 50, and alcohol consumption was assessed at the ages of 20 and 40, this may introduce some degree of recall bias and misclassification. However, this is a common limitation in retrospective studies examining the relationship between early-life exposure to environmental factors and disease course later in life. Any recall inaccuracy is likely similar across participants, meaning that it is unlikely to have systematically influenced the observed associations. Second, we only used a single brain age prediction method in this study. While brainageR is one of the most widely used methods,^46^ other models may yield different results. Third, we did not apply multiple-testing corrections. However, we treated analyses as exploratory rather than as definitive confirmatory tests. Applying conservative corrections would have reduced power and increased risk of false negatives, potentially obscuring real associations of interest.^47^ In addition, because pwMS who underwent a home visit had higher EDSS scores than those completing the full-day visit, our cohort may be slightly biased toward individuals with milder disease. Nevertheless, our findings remain informative, because the hospital group included the full range of disability. Last, we did not explore the effect modification according to MS subtypes, because of limited sample sizes, which would lead to insufficient statistical power.

To conclude, DMFs unrelated to MS, particularly smoking and alcohol consumption, contribute to neurodegenerative damage, leading to older looking brains. This neurodegenerative process contributes to worse disability and performance on cognitive tests. This study offers new insights into how DMFs may influence neurodegeneration and functional outcomes in MS, highlighting the potential of lifestyle interventions to mitigate neurodegeneration and ultimately disability progression.

## Glossary

3D: 3-dimensional
9HPT: 9-hole peg test
BPF: brain parenchymal fraction
brain-PAD: brain-predicted age difference
DAG: directed acyclic graph
DMF: disease-modifying factor
DMT: disease-modifying treatment
EDSS: Expanded Disability Status Scale
FLAIR: fluid-attenuated inversion recovery
GPR: Gaussian Process Regression
HC: healthy control
MACFIMS: Minimal Assessment of Cognitive Function in MS
MS: multiple sclerosis
pwMS: people with MS
T1w: T1-weighted
T2LV: T2 lesion volume
T25FWT: timed 25-foot walking test
